# Systemic administration of heparin ameliorates radiation-induced oral mucositis—preclinical studies in mice

**DOI:** 10.1007/s00066-018-1300-8

**Published:** 2018-04-16

**Authors:** Maria Kowaliuk, Eva Bozsaky, Sylvia Gruber, Peter Kuess, Wolfgang Dörr

**Affiliations:** 1 Department of Radiotherapy—ATRAB—Applied and Translational Radiobiology and Christian Doppler Laboratory for Medical Radiation Research for Radiation Oncology, Medical University of Vienna, Waehringer Gürtel 18–20, 1090 Vienna, Austria; 2 Department of Radiotherapy—Christian Doppler Laboratory for Medical Radiation Physics for Radiation Oncology, Medical University of Vienna, Vienna, Austria

**Keywords:** Oral mucositis, Normal tissue effects, Heparin, Radiotherapy, Mouse, Orale Mukositis, Normalgewebereaktionen, Heparin, Radiotherapie, Maus

## Abstract

**Purpose:**

The present study investigates the impact of systemic application of heparins on the manifestation of radiation-induced oral mucositis in a well-established mouse model.

**Materials and methods:**

Male C3H/Neu mice were irradiated with either single-dose or fractionated irradiation protocols with 5 × 3 Gy/week, given over one (days 0–4) or two (days 0–4, 7–11) weeks. All fractionation protocols were concluded by a local test irradiation (day 7/14) using graded doses to generate complete dose–effect curves. Daily doses of unfractionated or low molecular weight heparin (40 or 200 I.U./mouse, respectively) were applied subcutaneously over varying time intervals. The incidence and the time course of mucosal ulceration, corresponding to confluent mucositis in patients (RTOG/EORTC grade 3), were analysed as clinically relevant endpoints.

**Results:**

Systemic application of heparins significantly increased the iso-effective doses for the induction of mucosal ulceration, particularly in combination with fractionated irradiation protocols. Moreover, a tentative prolongation of the latent time and a pronounced reduction of the ulcer duration were observed.

**Conclusion:**

These data provide the first evidence for a protective and/or mitigative effect of heparins for radiation-induced oral mucositis. Further studies are ongoing investigating the underlying mechanism.

**Electronic supplementary material:**

The online version of this article (10.1007/s00066-018-1300-8) contains supplementary material, which is available to authorized users.

## Zusammenfassung

**Ziel:**

Die vorliegende Studie untersucht den Effekt der systemischen Gabe von Heparin auf die Ausprägung der radiogenen oralen Mukositis in einem etablierten Mausmodell.

**Material und Methoden:**

Männliche C3H/Neu-Mäuse wurden wie folgt bestrahlt: Einzeldosen oder fraktionierte Bestrahlungen mit 5 × 3 Gy/Woche über eine (Tage 0–4) oder zwei Wochen (Tage 0–4, 7–11). Bei allen fraktionierten Bestrahlungsprotokollen folgte eine lokale Aufsättigungsbestrahlung mit gestaffelten Dosen (Tag 7/14), um komplette Dosis-Effekt-Kurven zu generieren. Die tägliche Behandlung mit nichtfraktioniertem oder niedermolekularem Heparin (40 bzw. 200 I.E./Maus) erfolgte subkutan über variierende Zeitintervalle. Die Inzidenz und der zeitliche Verlauf der Schleimhautulzeration, entsprechend der konfluenten Mukositis in Patienten (RTOG/EORTC Grad 3), wurden als die klinisch relevanten Endpunkte analysiert.

**Ergebnisse:**

Die systemische Behandlung mit Heparin führte zu einer deutlichen Erhöhung der isoeffektiven Aufsättigungsdosen, besonders in Kombination mit fraktionierter Bestrahlung. Des Weiteren wurden eine tendenzielle Verlängerung der Latenzzeit und eine deutliche Verkürzung der Ulzerationsdauer beobachtet.

**Schlussfolgerung:**

Die vorliegende Studie liefert die ersten Hinweise auf einen protektiven bzw. mildernden Effekt von Heparin auf die Ausprägung der radiogenen oralen Mukositis. Weitere Studien wurden initiiert, um die zugrundeliegenden Mechanismen zu untersuchen.

## Background

Oral mucositis is a frequent early side effect of radio(chemo)therapy of head and neck tumours. Virtually all patients undergoing curative treatment develop oral mucositis, with more than 50% of the patients developing severe, confluent mucositis (grade 3–4 of the established scoring systems; [1]). Although oral mucositis has a major impact on the patient’s quality of life and tumour control probability, no biology-based prophylactic or mitigative approach has so far found entrance into clinical practice. Currently, supportive care strategies are purely symptomatic, based on the improvement of oral hygiene and pain relief [2].

The early radiation response of the oral mucosa is a complex process, based on the impairment of cell proliferation in the germinal epithelial layer [3]. This results in epithelial hypoplasia and consequently in complete denudation. These events are preceded and accompanied by vascular and inflammatory processes. The expression of several pro-inflammatory molecules—such as tumour necrosis factor-α (TNF-α), interleukins 1 and 6 (IL-1/6), nuclear factor-κ B (NF-κB), cyclooxygenase-2 (COX-2), platelet factor 4 (PF4) and matrix metalloproteinase (MMPs) among others—is modulated after irradiation.

Heparins are highly sulphated anionic polysaccharides, which have been used for over eighty years in the clinic due to their anticoagulant activity to prevent thrombosis [4]. Currently, a great body of literature is emerging proposing various binding partners for heparins, including growth factors, extracellular matrix (ECM) proteins, cytokines and chemokines regulating the processes of proliferation, development, inflammation, infection, wound healing and coagulation [5]. Therefore, the present study was initiated to investigate the muco-protective potential of heparins in a well-established mouse model of radiation-induced oral mucositis.

## Materials and methods

### Animals and housing

The experiments were performed in 12–14-week-old male mice of the inbred C3H/Neu strain from the breeding facility of the Department for the Biomedical Research of the Medical University of Vienna. Mice were bred and housed under specified pathogen-free conditions with controlled temperature (22 ± 2 °C), humidity (55 ± 10%) and a 12/12-h light–dark rhythm. Animals were provided with standard mouse diet (Sniff Spezialdiäten GmbH, Soest, Germany) and filtered water ad libitum. A maximum of five animals were housed in IVC cages (Tecniplast®, Buguggiate, Italy) on aspen wood bedding (ABEDD®, Lab & Vet Service GmbH, Köflach, Österreich).

### Irradiation technique

The irradiation technique of the murine oral mucosa has been described in detail elsewhere [6]. In brief, two different techniques were used: single-dose irradiation given to a 3 × 3 mm^2^ test area of the lower tongue surface or daily fractionated irradiation (5 × 3 Gy/week) given to the whole snouts of mice. The fractionated irradiation protocols were followed by local irradiation of the test area at the lower tongue using graded doses in order to generate complete dose–effect curves. Both protocols were performed using an YXLON MG325 X‑ray device (YXLON International GmbH, Hamburg, Germany) with a vertical beam.

For single-dose and local test irradiation, the animals were anesthetized by systemic administration of pentobarbitone sodium (Release®, WDT eG, Garbsten, Germany; 60 mg/kg intraperitoneally) and placed in a supine position in an aluminium block (diameter 25 mm). The tongue was gently pulled through a hole on the upper side of the block and fixed to the block by means of adhesive tape. In order to prevent disturbed blood circulation in the tongue, the head of the animal was supported by a polystyrene wedge. To define the irradiation field, an aluminium plate with a 3 × 3 mm^2^ window was placed over the lower tongue. The rest of the tongue as well as the body of the animal was shielded by a lead equivalent. The X‑ray unit was operated with a tube voltage of 25 kV and a tube current of 20 mA. The dose rate at the focus-to-surface distance of 15 cm was approximately 3 Gy/min. In addition to the inherent filtration with 3 mm Be, a 0.3 mm Al beam filter was used.

For percutaneous irradiation of the whole snouts, the mice were guided into a Perspex tube (28 mm inner diameter). A conical hole at the front end of the tube served for the positioning of the animal. The back end of the tube was closed to prevent the animal from escaping. Up to eight mice were irradiated simultaneously. The snout of the animals, including the entire tongue, was irradiated; the rest of the body was shielded by a lead equivalent. The X‑ray unit was operated with a tube voltage of 200 kV and a tube current of 20 mA. The dose rate at the focus-to-surface distance of 45.5 cm was approximately 1 Gy/min. In addition to the inherent filtration with 3 mm Be, 4 mm Al and 0.6 mm Cu beam filters were used. The dose homogeneity between the individual snout positions was ±3%.

### Heparins

Unfractionated heparin (UFH; heparin medicamentum®, medicamentum pharma, Allerheiligen im Mürztal , Austria) and low-molecular-weight heparin (LMWH; enoxaparin sodium, Lovenox®, Aventis, Strasbourg, France) were diluted in saline and injected subcutaneously at a daily dose of 40 I.E/mouse and 200 I.E/mouse, respectively (injection volume 150 µL). On irradiation days, the drug was applied 2 hours after irradiation and on days without irradiation approximately at the same time of day, i.e. at 11 am.

### Experimental design

**Fig. 1:**
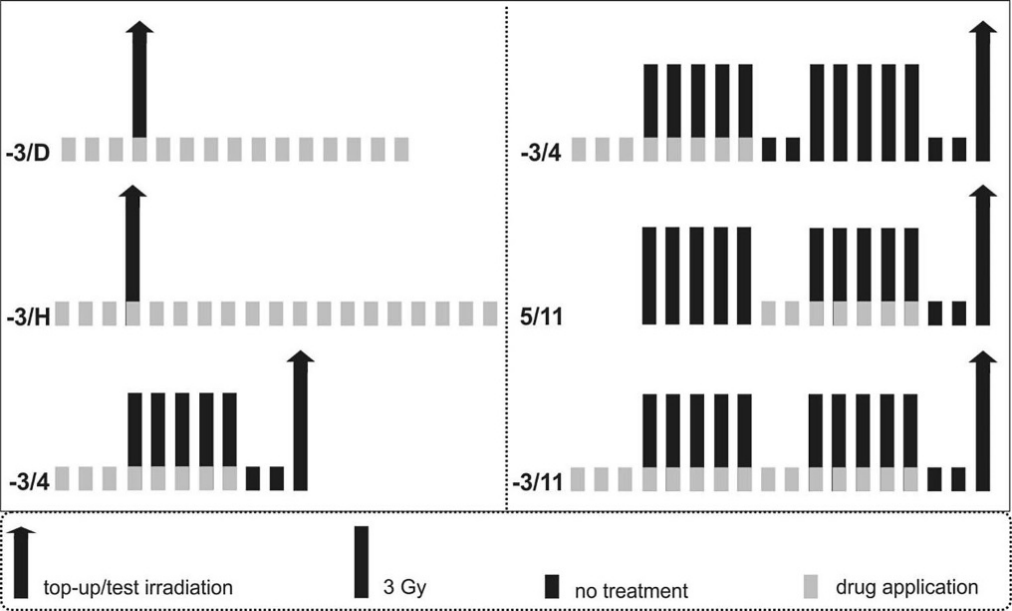
Experimental design. Animals were irradiated using either single-dose (**a**) or fractionated irradiation (**b**) protocols, concluded by a local test irradiation using graded doses. Irradiation was combined with varying drug application intervals, as indicated by the *light grey bars*. Each row represents one single experiment performed either with unfractionated or low-molecular-weight heparin. The labelling next to each experimental row indicates the first and last day of drug application. *D* diagnosis of the ulceration, *H* healing of the ulceration

Fig. 1 illustrates the schematic overview of the experimental design summarizing the irradiation and drug application protocols.

Single-dose irradiation was performed on day 0 using five graded dose groups. Each group comprised 10 animals (50 animals per experiment). UFH or LMWH was applied from 3 days before irradiation until either the first diagnosis (−3/D) or complete healing of the ulceration (−3/H). In non-responding animals, the drug application was stopped when all responding animals showed complete healing of the tongue ulcers.

Fractionated irradiation was given on 5 consecutive days per week over one (day 0–4) or two (days 0–4, 7–11) weeks and concluded by graded local top-up doses (5 dose groups, 10 animals each) on day 7 or 14, respectively; When fractionated irradiation was applied over 1 week, the drug was given 3 days before the first fraction until day 4. In combination with 2 weeks of fractionated irradiation, the drug was applied from day −3 until day 4 (−3/4) or day 5 to 11 (5/11) or day −3 until 11 (−3/11).

### Follow-up and endpoints

Mucosal ulceration within the test area of the lower tongue surface was identified from the first sign of the reaction until complete re-epithelization of the mucosa, i.e., for a maximum of 14 days. For this, scoring of the tongue was performed daily under anaesthesia with pentobarbitone sodium (Release®; 40 mg/kg intraperitoneally) under a cold light source, and the mucosal radiation response was documented. The incidence of ulceration served as primary endpoint. Time course parameters such as latency (time between top-up irradiation and first diagnosis of the ulceration) and ulcer duration (time between first diagnosis and complete healing) were analysed as secondary endpoints.

### Statistical analysis

Statistical analysis was performed using the SAS statistical software (SAS Institute Inc., Cary, NC, USA). To calculate complete dose–effect curves, probit analyses were performed under assumption of a log-normal distribution and no threshold dose. Dose–effect relationships were described by the ED_50_-values, representing the dose at which over 50% of animals are expected to show ulceration, and the corresponding standard deviation σ. To estimate the dose-dependence of the ulceration incidence, the p_dose_ was calculated based on the slope of the regression curve (probit analyses). To compare the dose–effect relationship of different experiments, a likelihood ratio test was used based on the logit model without a threshold dose.

## Results

An overview of the results is given in Table 1. Irradiation and heparin treatment were well tolerated by the animals. No treatment-associated adverse effects such as weight loss or food and behaviour changes were observed, with the exception of mucosal radiation response.

**Table 1:** Effect of systemic heparin application on the manifestation of oral radiation response

Drug application first–last day	Applied drug	ED50 ± SD (Gy)^a^	P_dose_^b^	P_control_^c^	Latency ± SD (d)^d^	Ulcer duration ± SD (d)
*Single-dose irradiation*						
–	–	11.8 ± 1.4	0.0004	–	10.8 ± 0.6	3.5 ± 0.6
−3–D	UFH	15.8 ± 4.2	0.0250	0.164	12.4 ± 0.5	3.5 ± 0.6
−3–H	UFH	18.3 ± 4.3	0.0113	0.0004	11.5 ± 0.5	3.4 ± 0.5
−3–D	LMWH	11.0 ± 0.7	0.0028	0.2857	11.5 ± 0.7	3.2 ± 0.7
−3–H	LMWH	13.1 ± 1.7	0.0008	0.1416	11.4 ± 0.8	3.0 ± 0.5
*Fractionated irradiation 5* *×* *3Gy/1 week*						
–	–	8.8 ± 1.4	0.0005	–	7.9 ± 0.6	4.0 ± 0.6
−3–4	UFH	15.4 ± 3.3	0.0050	<0.0001	7.8 ± 0.6	3.0 ± 0.7
−3–4	LMWH	11.6 ± 2.4	0.0016	0.0109	7.9 ± 0.7	3.6 ± 0.6
*Fractionated irradiation 10* *×* *3Gy/2 weeks*						
–	–	9.3 ± 1.8	0.0003	–	7.9 ± 0.6	4.6 ± 1.1
−3–4	UFH	19.1 ± 4.0	0.0158	<0.0001	7.2 ± 0.6	2.9 ± 0.6
−3–11	UFH	19.4 ± 6.2	0.0888	<0.0001	10.3 ± 0.8	2.6 ± 0.6
5–11	UFH	18.5 ± 3.5	0.0163	<0.0001	8.3 ± 0.7	3.0 ± 0.5
−3–4	LMWH	13.9 ± 2.9	0.0086	0.0012	8.8 ± 0.6	2.9 ± 0.6
−3–11	LMWH	17.7 ± 3.3	0.0209	<0.0001	9.5 ± 0.5	2.6 ± 0.5
5–11	LMWH	17.8 ± 3.4	0.0276	<0.0001	7.8 ± 0.9	2.8 ± 0.6

^a^Standard deviation of the ED_50_ values, calculated by logit analyses

^b^*p*-values for the radiation dose dependence of the ulcer incidence calculated by logit analyses

^c^*p*-values for the difference between the dose effect-curves of the control and the corresponding experiment, calculated by maximum likelihood analyses

^d^Time between the local/top-up irradiation and the first diagnosis of the ulceration

*UFH* unfractionated heparin, *LMWH* low-molecular weight heparin, *SD* standard deviation, *D* diagnosis of the ulceration, *H* healing of the ulceration

### Effect of heparin treatment in combination with single-dose irradiation

**Fig. 2:**
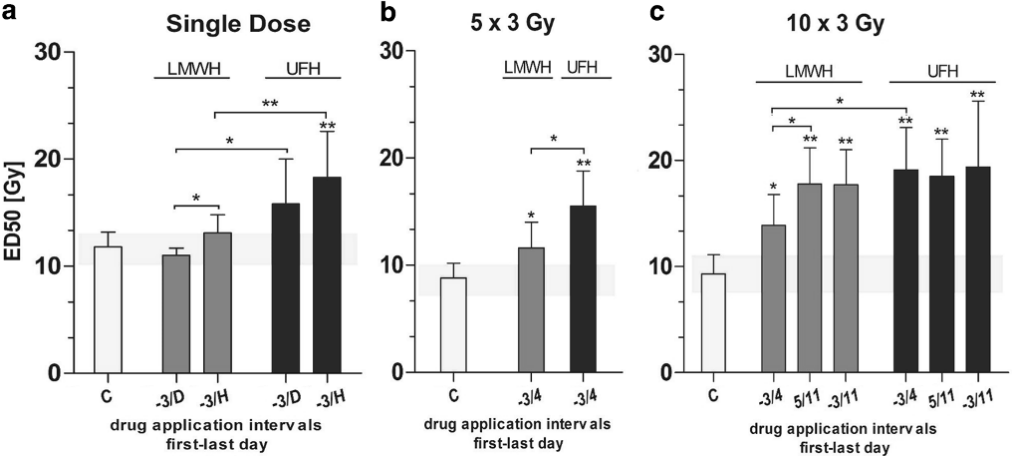
Effect of systemic heparin treatment on the ulcer incidence in combination with single-dose (**a**) or fractionated irradiation given over 1 (**b**) or 2 weeks (**c**). *Bars* represent the ED_50_ values of the test irradiation based on experiments with 5 graded dose groups with 10 animals each. ED_50_ values and the corresponding standard deviation σ (*error bars*) were calculated by logit analyses. The x‑axis represents varying drug application protocols. *Shaded area* displays the ED_50_ ± 1 SD of the corresponding control experiment (irradiation alone). *UFH* unfractionated heparin, *LMWH* low-molecular weight heparin, *C* control; *D* diagnosis of the ulceration; *H* healing of the ulceration. *p < 0.05, **p < 0.005

The iso-effective dose for the induction of mucosal ulceration for single-dose irradiation without drug application was 11.8 ± 1.4 Gy. Ulcer incidence was highly dose dependent (p_dose_ 0.0004). Systemic application of LMWH in combination with single-dose irradiation did not significantly increase the ED_50_ values (Table 1 and Fig. 2a) for either drug administration interval. As represented by the full dose–response curve, application of UFH from day − until the healing of the ulceration led to a significant shift of the iso-effective dose for the induction of mucosal response (Online Resource 1a). The ED_50_ value was 18.3 ± 4.3 Gy.

The mean latent time after the single-dose irradiation alone was 10.8 ± 0.6 d and the mean ulcer duration 3.5 ± 0.8 d. Application of heparins led to a tentative prolongation of the latent time, but the ulcer duration was not significantly changed.

### Effect of heparin treatment in combination with fractionated irradiation over 1 week

Fractionated irradiation over 1 week followed by top-up irradiation with graded doses led to an ED_50_ for the test irradiation of 8.8 ± 1.4 Gy. Irradiation in combination with heparin treatment resulted in significantly increased iso-effective doses for the induction of mucosal ulceration (Fig. 2b and Online Resource 1b). Particularly when UFH was applied, the increase of the ED_50_ value was highly significant (15.5 ± 3.3 Gy; *p* < 0.0001). The mean latent time after 1 week of fractionated irradiation was 7.9 ± 0.6 d and it took 4.5 ± 0.6 d for the ulceration to heal. Heparin application had no major impact on the latent time; the ulcer duration was slightly reduced, specifically when UFH was applied.

### Effect of heparin treatment in combination with fractionated irradiation over 2 weeks

The ED_50_ value for the test irradiation after 2 weeks of fractionated irradiation was 9.3 ± 1.8 Gy. Treatment with heparins led to a significant elevation of the ED_50_ values in combination with all drug application protocols (Fig. 2c). Particularly when UFH was applied, the shift of the ED_50_ values was highly significant (Online Resource 1c; *p* < 0.0001). Comparing the fractionated irradiation over 2 weeks in combination with drug application (LMWH or UFH) from day 3 until 4 (first irradiation week), treatment with UFH had a more pronounced impact on the iso-effective doses (*p* 0.0323).

The mean latent time after fractionated irradiation given over 2 weeks was 7.9 ± 0.6 d and the ulcer duration 4.6 ± 1.1 d. Heparin treatment clearly prolonged the latent time, particularly when UFH or LMWH was given over both weeks of irradiation (10.3 ± 0.8 d and 9.5 ± 0.5 d, respectively). Also, the ulcer duration was markedly reduced when heparins were applied from day −3 until 11 (2.6 ± 0.6 d for UFH and 2.6 ± 0.5 d for LMWH).

## Discussion

Oral mucositis induced by conventional radiotherapy in the head-and-neck region is a common and significant issue in oncology. Associated with severe pain, speaking and swallowing difficulties, oral mucositis has a severe impact on the patient’s quality of life. In addition, patients are at a high risk of local and systemic infections that may even result in septic events. These symptoms often lead to treatment interruptions, thus lowering the control probability of the head-and-neck tumours. Furthermore, the risk of developing consequential late side effects, such as dysphagia, chronic oesophageal infections, chronic or mucosal ulcerations or osteoradionecrosis increases with the duration and severity of the oral mucositis [7].

Many strategies, both prophylactic and therapeutic, have been proposed to manage the early oral radiation response. So far, however, no biology-based prophylactic or treatment approach has been generally introduced into clinical routine. Current supportive measures focus on pain and discomfort reduction, including improved oral hygiene, suitable diet, mouth washes, mucosal coating agents, cryotherapy, and local or systemic analgesia [2]. Therapeutic strategies tested pre-clinically and clinically include application of growth factors, normal tissue protection or scavenging of free oxygen radicals [8].

The clinical manifestation of radiation-induced oral mucositis is a dynamic and interactive event comprising multiple molecular and cellular processes, and is strongly associated with inflammatory changes in all compartments of the mucosa. Currently, a great body of literature is emerging proposing that some of those biological processes can be modulated by heparins, such as proliferation, infection, immune response, cell adhesion and inflammation [5]. Additionally, it appears that heparins, especially LMWH, exhibit an antitumour and antimetastatic potential [9]. Thus, this study was initiated to investigate the effect of two different heparin preparations on the manifestation of radiation-induced oral mucositis in the well-established and accepted mouse tongue model.

Systemic treatment with heparins significantly affected the manifestation of radiation-induced oral mucositis, with a significantly lower incidence of mucosal ulceration; the latent time was clearly prolonged and the ulcer duration reduced. These effects became particularly prominent when heparin application was combined with fractionated irradiation over 2 weeks.

Taking into account the α/β value of the oral mucosa (α/β = 11.6 Gy, [21]), it is possible to calculate the number of fractions compensated by the heparin treatment [10]. UFH application led to a compensation of 5.0 ± 3.2 fractions of 3 Gy. The maximum number of 3 Gy fractions compensated by LMWH treatment was 3.9 ± 1.0 (drug application over the second week of irradiation). These results imply a strong muco-protective effect of the systemic heparin application on the radiation response of the oral mucosa. Application of LMWH and UFH in combination with fractionated irradiation over 1 week led to a compensation of 0.9 ± 0.4 and 2.8 ± 1.1 fractions of 3 Gy.

The biological mechanisms behind the muco-protective effect of heparins are still not clear. As described in the literature, oral mucositis is associated with the activation of NF-κB and a subsequent upregulated expression of pro-inflammatory molecules, e.g. TNF α and IL-1/6 [3]. Several preclinical and clinical studies demonstrated that increased levels of these cytokines in the epithelium correlated with the development and severity of oral mucositis. In experiments using endothelial cells it was shown that heparin treatment inhibited the transcriptional activity of NF-κB [11, 12]. Furthermore, as demonstrated by Spratte et al, treatment with LMWH inhibited TNF-α signalling in human endometrial stromal cells in an NF-κB dependent manner [13]. Additionally, they showed that also the TNF-α induced overexpression of IL-6 was inhibited in the presence of heparin. Furthermore, recent studies suggest the involvement of deregulated expression of matrix metalloproteases (MMPs) in the pathobiology of mucositis [14]. A clinical study demonstrated that treatment with heparin reduced the plasma activity of MMPs [15].

Palifermin is an FDA-approved, “target-based” biologic agent for the prevention of oral mucositis. It is a human recombinant keratinocyte growth factor (KGF) which is expressed by fibroblasts and endothelial cells of different organs, typically in response to pro-inflammatory signals [16]. KGF expression stimulates proliferation of epithelial cells and modifies migration, differentiation and wound healing processes. Like other members of the fibroblast growth factor (FGF) family, KGF is a heparin-binding molecule and heparin is required for the maximum stimulation of KGF by its receptor [17]. For this reason, the mitigative effect of the heparin treatment observed in this study might be based on the increased stimulation of the KGF expression in the epithelium.

Adhesion of inflammatory cells to endothelial cells plays a role in the process of radiation-induced changes. As demonstrated previously by Jaal at al., expression of the endothelial intercellular adhesion molecule-1 (ICAM-1) was significantly increased during fractionated irradiation in the submucosa of a mouse tongue [18]. ICAM-1 is a cell surface protein present on a wide variety of cell types, such as fibroblasts, leukocytes, keratinocytes and endothelial cells, and, to some extent, the severity of the radiation-induced inflammatory reaction is associated with increased ICAM-1 levels [19]. As demonstrated by Lee at al., heparin treatment inhibited ICAM-1 expression at the transcriptional level in cerebral endothelial cells [12]. Since heparin cannot bind ICAM-1 directly, it is proposed that it affects the function of ICAM-1 indirectly by binding of macrophage antigen-1 (mac-1) that coordinates adhesive processes such as leucocyte migration [20]. The muco-protective properties of heparins might hence be due to the (indirect) interaction with this adhesion molecule.

Heparin showed the strongest effect when the treatment was combined with fractionated irradiation given over 2 weeks. In oral mucosa, there is a response to fractionated irradiation in the form of regenerative processes (“repopulation”) starting at the end of the first irradiation week and leading to increased radiation tolerance with the increasing overall irradiation time. As summarised by Dörr et al., repopulation comprises three major mechanisms: acceleration of stem cell proliferation, asymmetry loss of stem cell divisions and abortive divisions of sterilized cells [21]. The pronounced muco-mitigative effect of heparin application might be related to an interaction with any of these complex mechanisms. Potentially, the application of heparin leads to an earlier onset of the regenerative response, thus decreasing the incidence of ulcerations.

## Conclusion

The present study demonstrates for the first time a muco-protective potential of systemic heparin treatment for radiation-induced oral mucositis, particularly in combination with fractionated irradiation. Since both heparin preparations are already used in clinical settings, they seem to be promising drugs for future clinical studies. Nevertheless, mechanistic studies are necessary to elucidate the biological mechanisms underlying the modulatory effects of heparins (manuscript in preparation).

## Caption Electronic Supplementary Material

**Online Resource 1 Local test irradiation full dose–response curves for heparin effect on the radiation-induced oral mucositis after single-dose (a) and fractionated irradiation over one (b) or two (c) weeks. **Each curve represents the dose response of the test irradiation based on one experiment with 5 graded dose groups with 10 animals each, performed either with UFH or LMWH given over varying time intervals. Responders are defined as animals developing mucosal ulcerations. Shaded area displays the ED_50_ ± 1SD of the corresponding control experiment (irradiation alone). The corresponding ED_50_ and *p*-values are given in Table 1. *C* control; *D* first diagnosis of the ulceration; *H* healing of the ulceration.
