# Supplementary material for: Systemic administration of heparin ameliorates radiation-induced oral mucositis—preclinical studies in mice
Source: Strahlenther Onkol. 2018 Apr 16;194(7):686–92. doi: 10.1007/s00066-018-1300-8 (PMC6008356; doi:10.1007/s00066-018-1300-8)
Supplement: Supplementary file 1 — Online Resource 1 Local test irradiation full dose–response curves for heparin effect on the radiation-induced oral mucositis after single-dose (a) and fractionated irradiation over one (b) or two (c) weeks. Each curve represents the dose response of the test irradiation based on one experiment with 5 graded dose groups with 10 animals each, performed either with UFH or LMWH given over varying time intervals. Responders are defined as animals developing mucosal ulcerations. Shaded area displays the ED50 ± 1SD of the corresponding control experiment (irradiation alone). The corresponding ED50 and p-values are given in Table 1. C control; D first diagnosis of the ulceration; H healing of the ulceration. [file 66_2018_1300_MOESM1_ESM.pdf]

Ms. No. SUON-D-18-00031 Systemic administration of heparin ameliorates radiation-induced oral mucositis – preclinical studies in mice
Strahlentherapie und Onkologie
Maria Kowaliuk[1], Eva Bozsaky[1], Sylvia Gruber[1], Peter Kuess[2], Wolfgang Dörr[1]
[1]Medical University of Vienna, Department of Radiotherapy- ATRAB - Applied and Translational Radiobiology and Christian Doppler Laboratory for Medical Radiation Research for Radiation Oncology, Vienna, Austria
[2]Medical University of Vienna, Department of Radiotherapy- Christian Doppler Laboratory for Medical Radiation Physics for Radiation Oncology

Corresponding author:
Maria Kowaliuk
E-mail address: maria.kowaliuk@meduniwien.ac.at

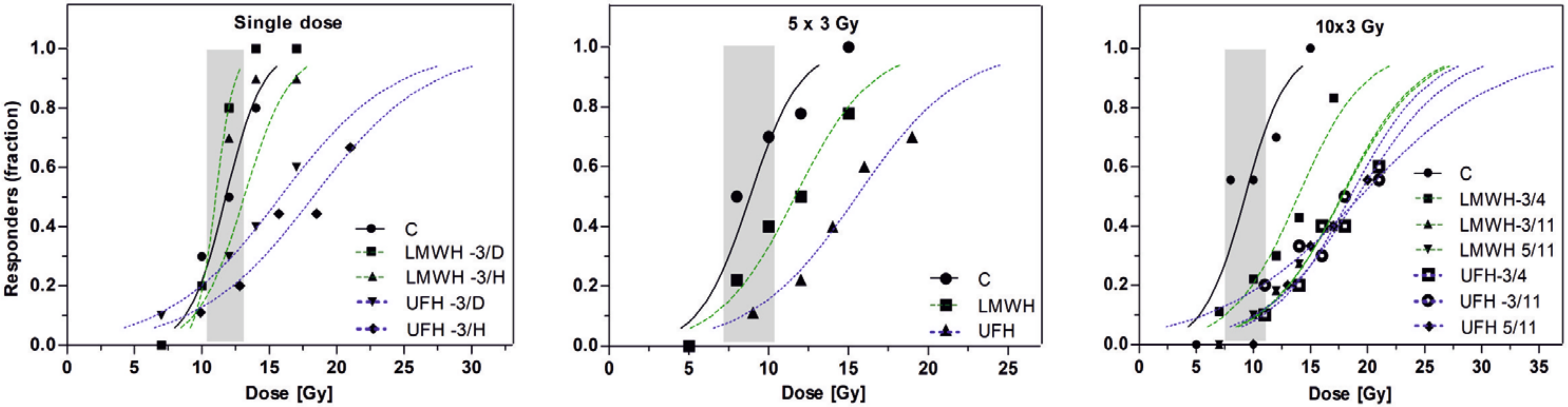

Online Resource 1 Local test irradiation full dose-response curves for heparin effect on the radiation-induced oral mucositis after single dose (a) and fractionated irradiation over one (b) or two (c) weeks.

Each curve represents the dose response of the test irradiation based on one experiment with 5 graded dose groups with 10 animals each, performed either with UFH or LMWH given over varying time intervals. Responders are defined as animals developing mucosal ulcerations. Shaded area displays the ED50 ± 1SD of the corresponding control experiment (irradiation alone). The corresponding ED_{50} and p-values are given in Table 1. C: control; D: first diagnosis of the ulceration; H: healing of the ulceration.
